# Baseline C-reactive Protein as a Risk Factor for Cryptococcal Meningitis and Death in HIV-associated Cryptococcal Antigenemia With CrAg Titer as an Effect Modifier

**DOI:** 10.1093/ofid/ofae392

**Published:** 2024-07-08

**Authors:** Caleb P Skipper, Paul Kirumira, Biyue Dai, Abduljewad Wele, Rose Naluyima, Teopista Namuli, Fred Turya, Patrick Muhumuza, Freddie Kibengo, David R Boulware, David B Meya, Elizabeth Nalintya, Radha Rajasingham

**Affiliations:** Infectious Diseases Institute, College of Health Sciences, Makerere University, Kampala, Uganda; Division of Infectious Diseases and International Medicine, Department of Medicine, University of Minnesota, Minneapolis, Minnesota, USA; Infectious Diseases Institute, College of Health Sciences, Makerere University, Kampala, Uganda; Division of Biostatistics & Health Data Science, School of Public Health, University of Minnesota, Minneapolis, Minnesota, USA; Division of Biostatistics & Health Data Science, School of Public Health, University of Minnesota, Minneapolis, Minnesota, USA; Infectious Diseases Institute, College of Health Sciences, Makerere University, Kampala, Uganda; Infectious Diseases Institute, College of Health Sciences, Makerere University, Kampala, Uganda; Infectious Diseases Institute, College of Health Sciences, Makerere University, Kampala, Uganda; Masaka Field Station, Medical Research Council/Uganda Virus Institute & London School of Hygiene and Tropical Medicine Uganda Research Unit, Masaka, Uganda; Masaka Field Station, Medical Research Council/Uganda Virus Institute & London School of Hygiene and Tropical Medicine Uganda Research Unit, Masaka, Uganda; Division of Infectious Diseases and International Medicine, Department of Medicine, University of Minnesota, Minneapolis, Minnesota, USA; Infectious Diseases Institute, College of Health Sciences, Makerere University, Kampala, Uganda; Division of Infectious Diseases and International Medicine, Department of Medicine, University of Minnesota, Minneapolis, Minnesota, USA; Infectious Diseases Institute, College of Health Sciences, Makerere University, Kampala, Uganda; Division of Infectious Diseases and International Medicine, Department of Medicine, University of Minnesota, Minneapolis, Minnesota, USA

**Keywords:** c-reactive protein, cryptococcal antigenemia, cryptococcal antigen titers, cryptococcal meningitis, HIV

## Abstract

**Background:**

Persons with HIV and cryptococcal antigenemia are at high risk of progression to cryptococcal meningitis or death. Baseline cryptococcal antigen (CrAg) plasma titer ≥1:160 is a known risk factor for poor outcomes, but other risk factors are unknown. In HIV-associated cryptococcal meningitis, baseline serum C-reactive protein (CRP) concentrations are positively associated with increased mortality. We hypothesized that CRP might also be associated with meningitis or death in persons with cryptococcal antigenemia.

**Methods:**

We measured plasma CrAg titers and CRP concentrations on cryopreserved serum from prospectively enrolled persons with HIV and cryptococcal antigenemia. Using time-to-event analyses, we compared 24-week meningitis-free survival in persons with normal CRP (<8 mg/L) and elevated CRP (≥8 mg/L). Logistic regression was used to assess how CRP concentration and CrAg titer might interact as covariates.

**Results:**

Of the 94 persons with elevated CRP, 19 (20.2%) developed meningitis or death, whereas of the 88 persons with normal CRP, 8 (9.1%) developed meningitis or death (*P* = .035). Persons with CrAg titer <1:160 and normal CRP had an ∼5% (3/61) event rate, whereas those with CrAg titer <1:160 but elevated CRP had an ∼20% (12/59) event rate. Importantly, we identified a statistically significant interaction effect between CrAg titer and CRP groups, in which elevated CRP increased risk in the low CrAg titer group (odds ratio, 1.54; 95% confidence interval, 1.16–2.04), but this effect was not present in high CrAg titer group (odds ratio, 0.78; 95% confidence interval, .53–1.15).

**Conclusions:**

Our findings demonstrate that CrAg titer may modify the direction of effect of CRP with meningitis-free survival; future studies should account for this interaction.

Cryptococcal meningitis is the most common cause of meningitis among persons with HIV in Africa, more common than bacterial meningitis [1, 2]. Despite widespread adoption of HIV test and treat programs and expanded access to antiretroviral (ART) medications, mortality rates from meningitis range from 25% in clinical trial settings up to 70% in typical community care settings, with most deaths occurring within months of diagnosis [3–5]. Cryptococcal antigen (CrAg) can be detected in the blood before onset of meningitis, and increasing CrAg titer predicts meningitis and death [6–8]. In Uganda, the national prevalence of cryptococcal antigenemia is 6%–8% in persons with CD4 < 100 cells/µL [8–11]. Fluconazole substantially improves survival among CrAg-positive persons, yet 25%–30% still develop meningitis or die despite this World Health Organization recommended antifungal therapy regimen [6–10, 12]. The identification of early predictors for those most likely to progress to meningitis or death among CrAg-positive persons would allow for triage to an enhanced level of care, such as more aggressive antifungal therapy or greater emphasis on diagnostic lumbar puncture [13].

The most established predictor for meningitis or death in CrAg-positive persons is baseline CrAg titer. Persons with CrAg lateral flow assay (LFA) titers ≥1:160 have a ∼3-fold higher risk of meningitis or death at 6 months in a Uganda study [10], and similar findings have been found in Ethiopia, South Africa, and Tanzania [13–15]. A few additional predictors have previously been identified including hyponatremia and lower body mass index [16, 17]. However, a thorough understanding of the landscape of risk predictors and how they interact with each other is needed to best design interventions to mitigate morbidity and mortality.

Serum C-reactive protein (CRP) concentrations at baseline have been identified as a risk factor for death in persons presenting with symptomatic HIV-associated cryptococcal meningitis [18]. CRP is a pentameric protein produced by the liver in response to inflammation—particularly by the cascading actions of interleukin-1 and interleukin-6—and activates the classical complement pathway [19]. In persons with HIV-associated cryptococcal meningitis, higher baseline serum CRP is associated with increased mortality (adjusted hazard ratio = 1.084 [95% confidence interval (CI), 1.031–1.139], per 10 mg/L increase) [18]; and persons identified as having CRP concentrations in the highest 2 quartiles had approximately 3-fold higher absolute mortality than persons with CRP concentrations in the lowest quartile.

Although previously published data show that elevated baseline CRP is a risk factor for death among people with cryptococcal meningitis, it is unknown whether CRP similarly predicts progression to cryptococcal meningitis and/or death among asymptomatic CrAg-positive persons. High CrAg titer is a strong prognostic factor that differentiates disease progression among people with cryptococcal antigenemia, yet it is unknown how CrAg titer might modify another potential risk factor such as CRP. The purpose of this study is to characterize the baseline CRP distribution among people with asymptomatic cryptococcal antigenemia and to evaluate whether elevated baseline CRP is a risk factor for meningitis or death. We further evaluated whether plasma CrAg titer could be a potential effect modifier.

## METHODS

We prospectively enrolled adults with HIV who were plasma CrAg-positive without symptoms of meningitis at baseline into a longitudinal observational cohort from 2016 to 2022 at 2 sites in Kampala and Masaka, Uganda. A subset of these participants were further enrolled into the ongoing ACACIA randomized clinical trial (Clinicaltrials.gov: NCT03945448). All participants received preemptive fluconazole, as recommended by the World Health Organization [20]. Some participants may have additionally received a single dose of 10 mg/kg liposomal amphotericin B. To protect the integrity of the ongoing parent trial, only blinded primary outcome data were accessible with approvals from the study's Data and Safety Monitoring Board and Trial Steering Committee. Therefore, there is no adjustment by randomized treatment arm in this analysis. Diagnoses were made using the CrAg LFA (IMMY; Norman, OK, USA) on plasma, and CrAg titers were determined using serial 2-fold dilutions. Participants who had any symptoms of meningitis at screening received a lumbar puncture to rule out cryptococcal meningitis. Persons who were cerebrospinal fluid CrAg LFA or fungal culture positive at CrAg screening were excluded from this analysis.

Cryopreserved serum from the time of study enrollment (baseline) were selected at convenience for CRP concentration measurement. Measurements occurred at the Infectious Diseases Institute (Kampala, Uganda) core laboratory if the sample was in Uganda; and correspondingly, at the University of Minnesota Medical Center (Minneapolis, Minnesota, USA) core laboratory for samples in Minnesota. Serum CRP was measured by reflectance spectrophotometry. All serum tested had no more than one previous freeze-thaw cycle. A subset of samples (N = 30) from the same participants were run at both sites (same blood draw, separate aliquot) to measure relative coefficient of variance between lab assays (Supplementary Methods). For samples that were run at both sites, the average CRP value for the paired samples was calculated and used in all analyses. At the Uganda laboratory, the lower limit of quantification of the assay was 0.5 mg/L. At the Minnesota laboratory, the lower limit of quantification was <3.0 mg/L. Thus, all values <3.0 mg/L were set to 3.0 mg/L for the analysis.

Baseline characteristics were summarized for the entire cohort. CRP groupings were determined using the prespecified reference range: normal CRP value (<8 mg/L) versus elevated CRP value (≥8 mg/L) [21]. Categorical variables were summarized with counts and proportions, and comparisons between the CRP subgroups were made using Fisher's exact test. Continuous variables were summarized with median and interquartile range, and comparisons between the subgroups were made using Kruskal-Wallis testing. The plasma CrAg titer subgroups (titer <1:160 vs titer ≥1:160) were prespecified, based on our prior pooled analysis [13], and we used these subgroups across all analyses.

The primary endpoint of interest was the development of meningitis or death within 24 weeks from study enrollment. Kaplan-Meier curves were used to characterize the time to development of meningitis or death within 24 weeks. Log-rank tests were conducted to compare the meningitis-free survival among the 2 CRP subgroups (normal vs elevated), and among 4 CRP and CrAg titer subgroups (normal CRP with low CrAg titer, normal CRP with high CrAg titer, elevated CRP with low CrAg titer, elevated CRP with high CrAg titer). Cox proportional hazard models were created for univariate assessment of predictors of interest (eg, CRP and CrAg titer) and for baseline characteristics found to be significantly skewed to 1 group (eg, sex, receiving ART). We estimated survival times by CRP group using restricted mean survival time, which use area under the survival curve.

To further evaluate the predictive performance of baseline CRP values and the relationship between CRP and CrAg titer, we log_2_-transformed CRP to model as a continuous variable. Univariate and multivariate logistic regressions were used to test association of independent variables against meningitis or death events modeled as a binary outcome. More specifically, to evaluate the potential effect modification from CrAg titers, an interaction term between CRP and CrAg titer was used in the multivariate model. Odds ratios and 95% CIs were estimated. Area under the curve (AUC) receiver operating characteristics curves were calculated for models with and without the interaction term between the log_2_-transformed CRP and CrAg titer subgroups. The relationship between the continuous log_2_-transformed CRP and the primary outcome of interest among each titer subgroup was visualized on the log odds scale. Confidence bands were also calculated to characterize whether the effect differs among titer subgroups. We conducted all statistical analyses using SAS version 9.3 (SAS Institute, Cary, NC) and R version 3.6.0 (R Core Team; Vienna, Austria).

The parent studies received approvals from both Uganda (Joint Clinical Research Center Institutional Review Board and Uganda National Council for Science and Technology) and University of Minnesota institutional review boards. All participants provided written informed consent, including for the storage of specimens for future research.

## RESULTS

A total of 182 people with HIV and asymptomatic cryptococcal antigenemia contributed to this analysis. The median age was 36 years (interquartile range, 30–42), and 55% (101/182) were male. Of these, 88 (48%) had a normal serum CRP (<8 mg/L), and 94 (52%) had an elevated CRP (≥8 mg/L) at baseline. Persons with elevated CRP were more likely to be male (64% vs 47%), had higher white blood cell counts (median 4100 vs 3600 per μL), and were less likely to be receiving ART at baseline (29% vs 51%) (Table 1). Importantly, CD4 count and CrAg titer were not different between groups. Overall, 60 persons (33%) had plasma CrAg titer ≥1:160.

**Table 1.** Baseline Characteristics by C-reactive Protein Grouping in Persons With HIV and Asymptomatic Cryptococcal Antigenemia

Baseline Characteristics	N With Data (N = 182)	CRP <8 mg/L (N = 88)	CRP ≥8 mg/L (N = 94)	*P* Value
Age, y	182	36 [30, 42]	37 [30, 42]	.88
Female	182	47 (53%)	34 (36%)	.02
Receiving ART	182	45 (51%)	27 (29%)	<.01
Duration of ART, mo	70	3.4 [.6, 8.0]	1.4 [0.3, 5.0]	.23
Fluconazole use in prior 14 d	172	42 (52%)	52 (56%)	.60
Receiving TB treatment	182	2 (2.3%)	3 (3.2%)	.71
Receiving cotrimoxazole	182	15 (17%)	13 (14%)	.55
CD4 count, cells/µL	154	50 [25, 96]	40 [17, 76]	.10
White blood cells, ×10^9^/L	176	3.6 [2.8, 4.5]	4.1 [2.9, 5.6]	<.01
Plasma CrAg titer ≥1:160	180	25 (29%)	35 (37%)	.25
Serum CRP, mg/L	182	≤3.0 [<3.0, 4]	34.5 [16.4, 65.0]	-

Values are median [interquartile range] or N (%). *P* values calculated by Kruskal-Wallis test for medians and Fisher exact tests for proportions.

Abbreviations: ART, antiretroviral therapy; CrAg, cryptococcal antigen; CRP, C-reactive protein; TB, tuberculosis.

**Figure 1.**
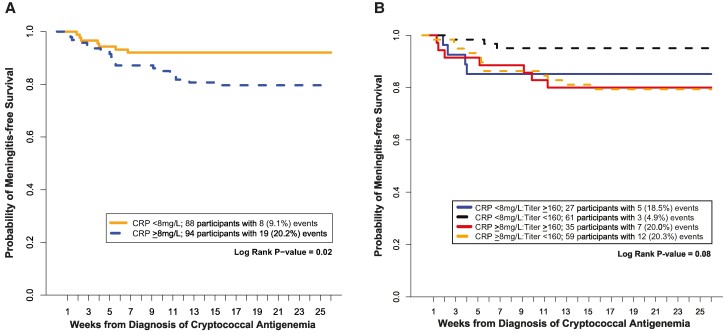
*A*, Cumulative meningitis-free survival at 24 wk by C-reactive protein grouping. Figure 1*A* demonstrates a Kaplan Meier curve for 24-wk meningitis-free survival by CRP grouping in persons with HIV and asymptomatic cryptococcal antigenemia. The primary event was the development of symptomatic meningitis or death. Elevated serum CRP (>8 mg/L) was associated with higher probability of meningitis or death as compared to a normal CRP (<8 mg/L). *B*, Cumulative meningitis-free survival at 24 wk by C-reactive protein and CrAg titer grouping. Figure 1*B* demonstrates a Kaplan Meier curve for 24-week meningitis-free survival by serum CRP and plasma CrAg titer grouping among those with HIV-associated cryptococcal antigenemia. Persons with normal CRP (<8 mg/L) and low CrAg titer (<1:160) had an event rate of ∼5%. Persons with elevated CRP (>8 mg/L) or high CrAg titer (>1:160) had an approximately 4-fold higher event rate of ∼20%. Although normal CRP had a protective effect among participants with low CrAg titer, this benefit was not observed among participants with high CrAg titer, where participants from both normal and elevated CRP groups experienced approximately the same event rate. Supplementary Table 1 breaks down the composite event outcome to either meningitis or death.

We compared 24-week meningitis-free survival by CRP groups. Of the 94 persons with elevated CRP, 19 (20.2%) developed meningitis or death, whereas of the 88 persons with normal CRP, 8 (9.1%) developed meningitis or death (*P* = .035) (Figure 1*A*). Elevated CRP (CRP ≥8 mg/L) was associated with greater risk of developing meningitis or death in a time-to-event analysis (hazard ratio = 2.70; 95% CI, 1.12–6.25; *P* = .026) (Table 2). Interestingly, in this small analysis cohort, risk for meningitis or death with CrAg titer ≥1:160 was not statistically significant by univariate analysis (hazard ratio = 1.58; 95% CI, .72–3.43; *P* = .25). The distribution of CRP concentrations by event status is visualized in Supplementary Figure 1.

**Table 2.** Univariate Proportional Hazards Regression for the Development of Meningitis or Death by 24 Weeks

Variable	Hazard Ratio	95% Confidence Interval	*P* Value
CRP ≥8 mg/L	2.70	1.12–6.25	.026
CRP, per log_2_ increase	1.20	1.00–1.46	.05
CrAg titer ≥1:160	1.58	.72–3.43	.25
Female	1.49	.69–3.22	.31
Receiving ART	0.78	.35–1.76	.55
White blood cells, ×10^9^/L	0.90	.70–1.15	.38

Table 2 displays hazard ratios and 95% confidence intervals. Serum CRP was associated with 24-week mortality when analyzed as either a dichotomous variable or when transformed into a log_2_ continuous variable. Plasma CrAg titer, female sex, receiving ART, and white blood cell count at baseline were not statistically significant for association with mortality as univariate predictors, although the possible effect size of CrAg titer is large enough to be of potential clinical relevance.

Abbreviations: ART, antiretroviral therapy; CrAg, CrAg, cryptococcal antigen; CRP, C-reactive protein.

To determine if the effect of elevated CRP was independent of CrAg titer (a known risk factor), we compared time-to-event outcome among the 4 CRP and CrAg titer subgroups. Persons with low CrAg titer and normal CRP had a much lower event rate of 4.9% than the event rates in the other 3 subgroups (Figure 1*B*; Supplementary Table 1). More specifically, persons with low CrAg titer but elevated CRP had a 20.3% event rate, similar to the event rates of the high CrAg titer groups, which did not differentiate by CRP subgroups among itself (18.5% vs 20.5%). We also reported the restricted mean survival time by 24 weeks for each group in Supplementary Table 2. The results are similar to the Kaplan-Meier curves in that the CRP <8 mg/L and CrAg titer <1:160 group have the longest event-free mean survival time of 23 weeks, whereas the other three groups have a mean survival time of approximately 20 weeks.

**Figure 2.**
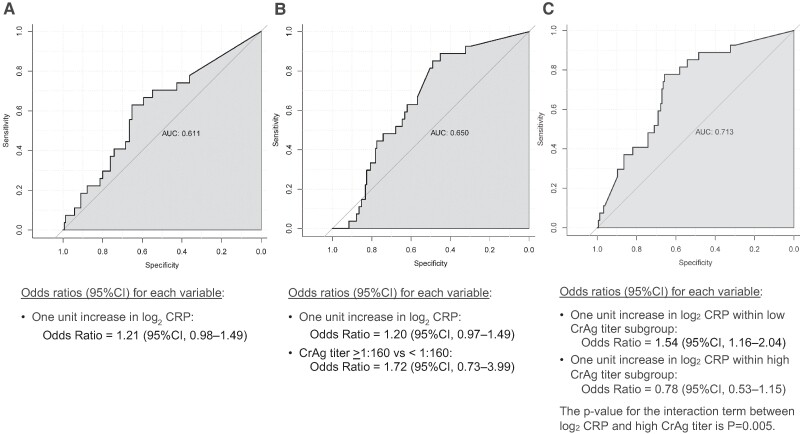
Logistic regression model measuring goodness of fit using area under the curve (AUC) for predicting future meningitis-free survival through 24 wk. (*A*) log2 CRP as lone predicator. (*B*) log2 CRP and CrAg titer >1:160. (*C*) log2 CRP and CrAg titer >1:160 with addition of an interaction term (log2 CRP * CrAg titer >1:160).

**Figure 3.**
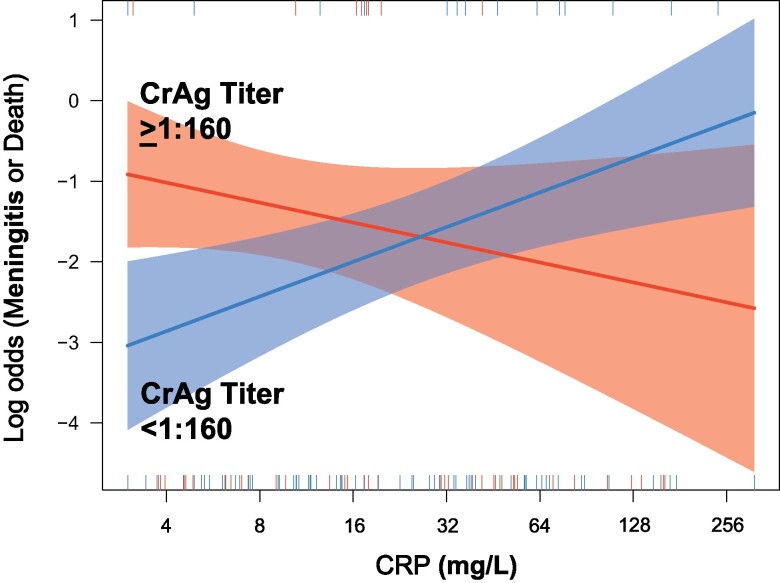
Log odds of clinical event per log2 CRP increase by CrAg titer grouping. Log odds of clinical events (meningitis or death) by increasing log_2_ CRP, stratified by CrAg titer grouping. Events are defined by progression to meningitis or death. In the group with CrAg titer <1:160, the log odds of an event increases as CRP increases. On the contrary, in the CrAg titer ≥1:160 group, the log odds of an event decreases as CRP increases. We speculate that this may indicate the immune system is still capable of generating an inflammatory response in the setting of systemic infection, where elevated CRP in low CrAg titer persons may represent undiagnosed AIDS-related pathology while elevated CRP in high CrAg titer persons may represent a commensurate immune response to a large burden of cryptococcal disease.

The same trend was observed when CRP was analyzed as a log_2_-transformed continuous variable in logistic regression models. Although both log_2_ CRP (odds ratio = 1.20; 95% CI, .97–1.49) and the high CrAg titer subgroup (odds ratio = 1.72; 95% CI, .73–3.99) were only modestly associated with the risk of meningitis or death in a multivariate logistic regression model (Figure 2), there was a significant interaction when both variables were combined with an interaction term between log_2_ CRP and CrAg titer group (*P* = .005). This indicates the association between CRP and the primary outcome differs between the low CrAg titer subgroup (odds ratio of log_2_ CRP = 1.54; 95% CI, 1.16–2.04) and the high CrAg titer subgroup (odds ratio of log_2_ CRP = 0.78; 95% CI, .53–1.15). The model including the interaction term also had a better fit (AUC = 0.713) than either the univariate CRP model (AUC = 0.611) or the multivariate model without an interaction term (AUC = 0.650) (Figure 2). Figure 3 visualizes the interaction on a log odds plot by CrAg titer subgroup.

## DISCUSSION

When investigating baseline CRP as a potential predictor for meningitis or death in persons with HIV-associated cryptococcal antigenemia, we evaluated an interesting and potentially important interaction between CRP and CrAg titer. We found that CRP ≥8 mg/L appeared to mitigate the protective effect of low CrAg titer in our population, with meningitis or death events rising from ∼5% in persons with CRP <8 mg/L and titer CrAg <1:160 to ∼20% in persons with CRP ≥8 mg/L and CrAg titer <1:160. Our subsequent logistic regression modeling suggested a significant interaction between CRP and CrAg titer. We determined that increasing CRP was significantly associated with *increased* risk of clinical events in the low CrAg titer group <1:160 (odds ratio = 1.54; 95% CI, 1.16–2.04), whereas the same effect was not found in the high CrAg titer group ≥1:160 (odds ratio = 0.78; 95% CI, .53–1.15), which instead saw a nonsignificant trend toward *decreased* events, when the model included an interaction term (*P* = .005). Although we recognize that the limitations of this study do not support declaring CRP as a predictor for meningitis or death in those with cryptococcal antigenemia, we do feel the observed pattern and covariate interaction are hypothesis generating and an important consideration for active researchers.

This surprising paradoxical response may have an immunological explanation. In persons with low CrAg titer (ie, low disseminated fungal burden), we hypothesize that elevated CRP may represent either (1) other undiagnosed opportunistic infections or AIDS-related pathology or (2) an overexuberant aberrant immune response resulting in host damage. In contrast, in persons with high CrAg titer (ie, high fungal burden), we hypothesize that elevated CRP represents a commensurate immune response for a significantly disseminated infection. Thus, there is scientific rationale for why CRP may be associated with worse outcomes in persons with low titers, yet better outcomes in persons with high titers; and these observations may be compatible with the damage response framework previously described in cryptococcosis [22, 23].

Importantly, we additionally demonstrated that including the interaction between CRP and CrAg titer improves the predictive model fit better than a model with the same covariates but without the interaction term. Overall, this suggests that this heterogeneity between the high-titer and low-titer subgroups should be considered for inclusion in future predictive models of CrAg titer or CRP.

Although these findings of effect modification of CrAg titer are consistent across various modeling approaches, the result is still limited by the number of observations. As is noted in Figure 3, the confidence band is wide in the region beyond a log_2_-transformed CRP of 5 (ie, 32 mg/L), indicating the number of participants with high CrAg titer and elevated CRP are few; therefore, the results need to be confirmed by larger studies. Similarly, we see a large effect size for the risk of CrAg titer ≥1:160 and meningitis or death, but a CI that crosses 1, further supporting that our sample size was small because this is a highly established risk factor. For pragmatic reasons, we quantified CRP concentrations from 2 different laboratory sites, which increases the interassay variability introduced. However, we were able to demonstrate highly correlated findings between the 2 sites by running a subset of paired samples. Missing data were more prevalent among certain variables (eg, baseline CD4 count) compared with other variables. Because the ACACIA trial is ongoing, outcome data stratified by intervention arm subgroup are not available for analysis to protect the integrity of the randomized trial. Last, we have not presented a full multivariate model of all the predictors of interest—which is planned for a future manuscript with a larger sample size. Ideally, a comprehensive risk factor analysis would account for expanded baseline laboratory parameters, differences in antifungal treatment, systemically screened concurrent infection, and HIV virologic factors. The purpose of this simplified manuscript is to highlight the important interaction between CRP and CrAg titer, particularly for those studying populations at risk for cryptococcal antigenemia.

In conclusion, we identified that elevated serum CRP initially appears to be associated with the development of meningitis or death in persons with HIV and asymptomatic cryptococcal antigenemia. However, on deeper investigation, we discovered an important interaction between CRP concentration and CrAg titer. This interaction complicates a simplistic interpretation of CRP or CrAg titer as a risk factor for poor outcomes in this population. Rather, our data suggest that increasing CRP is associated with an increased risk of meningitis or death in persons with CrAg titer <1:160, whereas an inverse trend is seen in persons with CrAg titer ≥1:160. These patterns of observation are valuable, but we posit that the limitations of this study make it difficult to declare CRP a predictor for meningitis or death in cryptococcal antigenemia. Instead, we advocate that accounting for this interaction in statistical models is important for future analyses. More studies are needed to better understand this interaction with CrAg titer and the overall prognostic value of CRP in this population.

## Supplementary Data

Supplementary materials are available at *Open Forum Infectious Diseases* online. Consisting of data provided by the authors to benefit the reader, the posted materials are not copyedited and are the sole responsibility of the authors, so questions or comments should be addressed to the corresponding author.

## Supplementary Material

ofae392_Supplementary_Data

## Notes

***Acknowledgments.*** We thank members of the ACACIA trial team and the participants.

***Data availability.*** A deidentified dataset can be made available on request to the corresponding author.

***Patient consent statement***. Written informed consent was obtained from all participants included in this study. Appropriate regulatory approvals were obtained from all relevant entities for this research (refer to last paragraph of Methods).

***Financial support*.** The study was supported by the National Eye Institute and the Fogarty International Center (R21EY035567); the National Center for Advancing Translational Sciences (KL2TR002492 and UL1TR002494); and the National Institute of Allergy and Infectious Diseases (K23AI138851, U01AI125003).
