# Supplementary material for: Baseline C-reactive Protein as a Risk Factor for Cryptococcal Meningitis and Death in HIV-associated Cryptococcal Antigenemia With CrAg Titer as an Effect Modifier
Source: Open Forum Infect Dis. 2024 Jul 8;11(8):ofae392. doi: 10.1093/ofid/ofae392 (PMC11288378; doi:10.1093/ofid/ofae392)
Supplement: ofae392_Supplementary_Data [file ofae392_supplementary_data.docx]

**Supplemental Methods**

We tested inter-assay concordance of 30 paired samples that were run at both the University of Minnesota and the Infectious Diseases Institute laboratories. The mean for the total paired samples was 31.3mg/L (SD, 49.3) at the University of Minnesota and was 33.4mg/L (SD, 43.2) at the Infectious Diseases Institute. We calculated the coefficient of variance (CV) by dividing the standard deviation by the mean and multiplying by 100. For the University of Minnesota, the CV was 1.58. For the Infectious Diseases Institute, the CV was 1.29. By Levene’s Test for Homogeneity of Variance of the mean, there was no difference between the two groups (p=0.73). The relationship between mean values of the two labs is visualized in the scatterplot below.

Scatterplot of raw CRP values by performing laboratory.


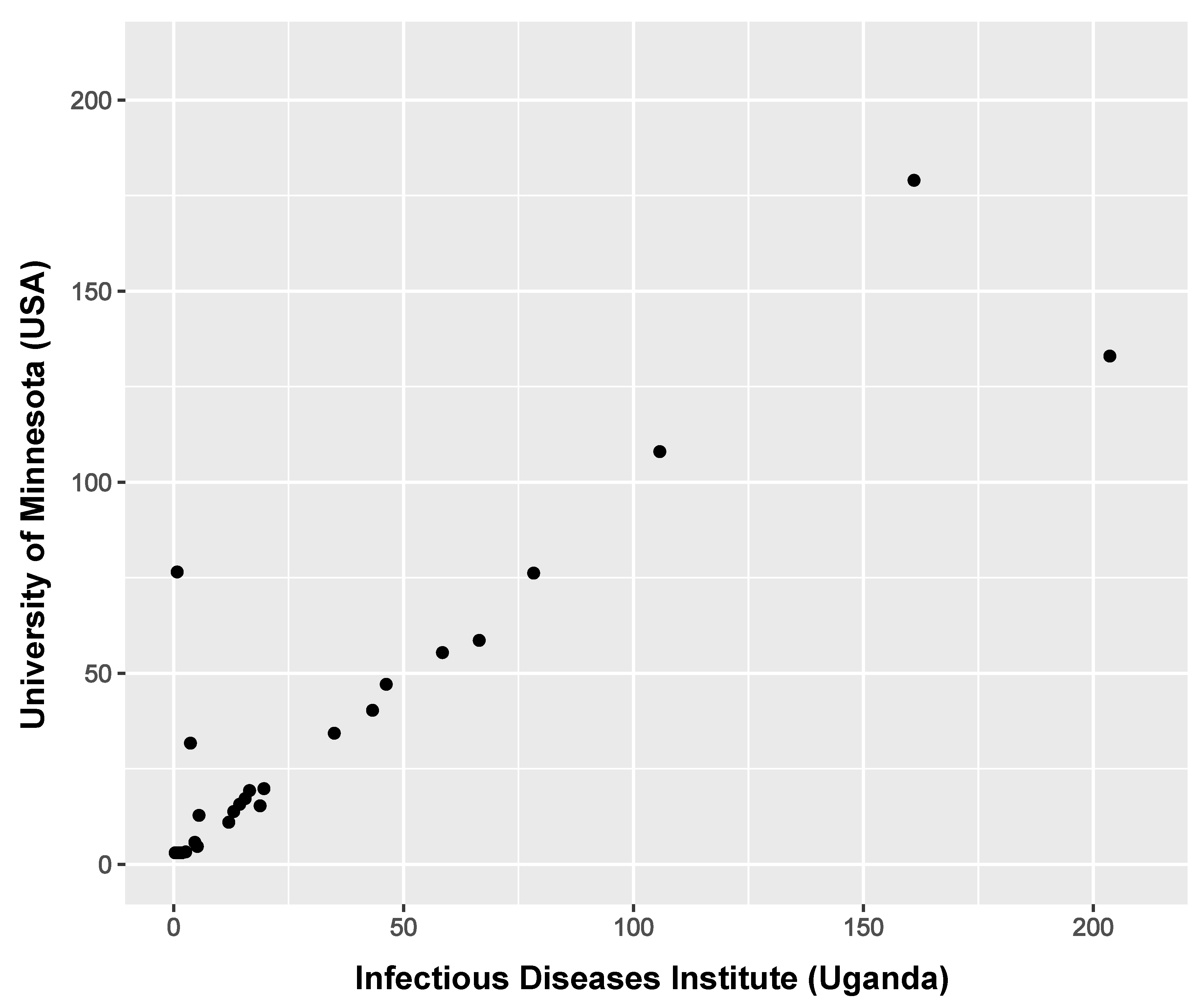


Overall, the concordance of the CRP measurement between the University of Minnesota laboratory and the Infectious Diseases Institute laboratory were high. We did note two significant outliers in the group. Both outliers had a normal CRP at the Uganda site, but an elevated CRP at the USA site. Upon further review, both of these samples had an ~24 hour difference in collection time, with the USA-run sample being the collected later compared to the Uganda-run sample. Thus, normal physiological changes could potentially explain the difference. As the average value was used in the analysis of paired samples, both of these participants were considered to have “elevated CRP”.

**Supplementary Table 1.** Events broken down by meningitis or death by CRP group.

| Event Breakdown | Low CRP (<8mg/L)  N=88 | | High CRP (>=8mg/L)  N=94 | | Overall |
| --- | --- | --- | --- | --- | --- |
|  | High titer | Low titer | High Titer | Low Titer |  |
| No event | 22 | 58 | 28 | 47 | 155 |
| Meningitis, survived | 3 | 1 | 2 | 0 | 6 |
| No Meningitis, but death | 0 | 2 | 5 | 11 | 18 |
| Meningitis, then death | 2 | 0 | 0 | 1 | 3 |
| Overall | 27 | 61 | 35 | 59 | 182 |

**Supplementary Table 2.** 24-week restricted mean survival time by CRP and CrAg titer subgroups.

| **By CRP Groups** | **Restricted Mean Survival Time**  **(weeks)** | | | **95% Confidence Interval** | **P-value** |
| --- | --- | --- | --- | --- | --- |
| Low CRP (<8mg/L) | 22.4 | | | (21.3, 23.5) |  |
|  |  | | |  |  |
| High CRP (>=8mg/L) | 20.4 | | | (18.9, 21.9) |  |
|  |  | | |  |  |
| Difference (Low CRP  - High CRP) | 1.97 | | | (0.1, 3.8) | 0.040 |
|  |  | | |  |  |
| **By CrAg Titer Groups** | **Restricted Mean Survival Time**  **(weeks)** | | | **95% Confidence Interval** | **P-value** |
| Low CrAg Titer (<1:160) | 21.8 | | |  |  |
|  |  | | |  |  |
| High CrAg Titer (>=1:160) | 20.5 | | |  |  |
| Difference (Low CrAg Titer – High CrAg Titer) | 1.3 | | | (-0.8, 3.5) | 0.225 |
| **By CRP and CrAg Groups** | **Restricted Mean Survival Time**  **(weeks)** | | | **95% Confidence Interval** |  |
| CRP <8mg/L and Titer <160 | 23.1 | | | (22.1, 24.0) |  |
| CRP <8mg/L and Titer >=160 | 20.6 | | | (17.6, 23.6) |  |
| CRP >8mg/L and Titer >=160 | 20.3 | | | (17.8, 22.8) |  |
| CRP >8mg/L and Titer <160 | 20.5 | | | (18.6, 22.3) |  |
|  | |  |  | |  |

**Supplemental Figure 1.** Violin plot of the distribution of CRP by meningitis or death.

**
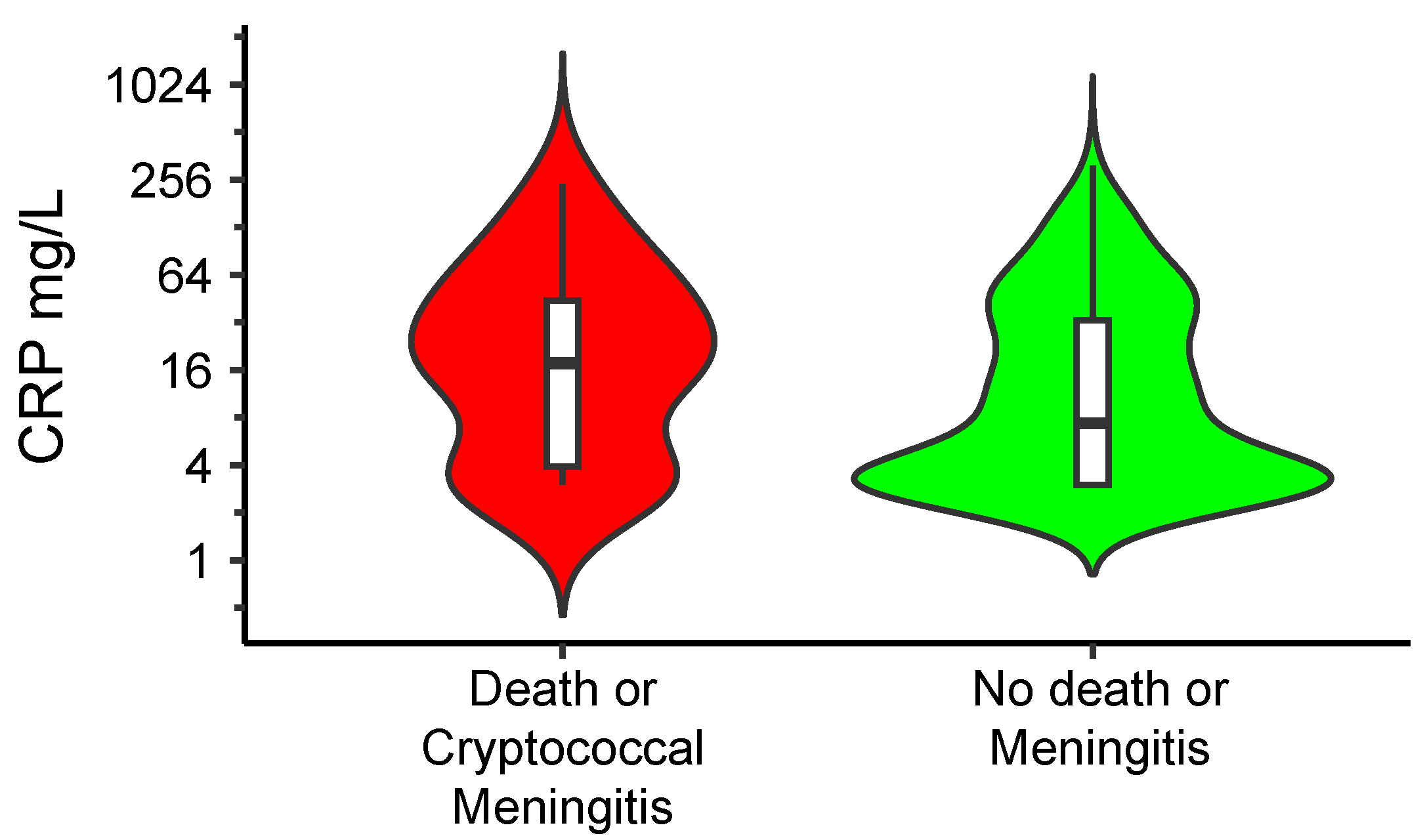
**

Supplemental Figure 1 demonstrates the distribution of CRP values on a log_2_ scale when grouped by participants who experienced a meningitis or death event, versus those without an event.
